# Heat and Moisture Transfer Depending on 3D-Printed Thermoplastic Polyurethane and Ethylene-Vinyl Acetate Foam and the Presence of Holes for 3D Printing Clothing Development

**DOI:** 10.3390/polym16121684

**Published:** 2024-06-13

**Authors:** Sunghyun Kwon, Sungeun Kwon, Heeran Lee, Murali Subramaniyam

**Affiliations:** 1 Seckinger High School, Buford, GA 30519, USA; seank7011@gmail.com (S.K.); ellie.kwon0204@gmail.com (S.K.); 2 Textiles, Merchandising and Interiors, The University of Georgia, Athens, GA 30602, USA; heeran.lee@uga.edu; 3 Department of Materials Design Engineering, Kumoh National Institute of Technology, Gumi 39177, Republic of Korea; 4 Department of Mechanical Engineering, Faculty of Engineering and Technology, SRM Institute of Science and Technology, Kattankulathur, Chennai 603203, India

**Keywords:** 3D-printed TPU, EVA foam, size of holes, materials, heat transfer, moisture transfer, clothing comfort

## Abstract

Recently, clothing development 3D printing and the evaluation of its physical characteristics have been explored. However, few studies have tackled thermal comfort, which is a major contributor to the wearers’ comfort. Therefore, this study was designed to suggest effective materials and hole sizes for clothing obtained by 3D printing to maintain a comfortable clothing environment. In particular, two main variables, namely five different materials and three-hole sizes, were analyzed. All samples were placed on a hot plate (36 °C), and their surface temperature and humidity were measured for 10 min. The samples with only thermoplastic polyurethane (TPU) achieved the largest temperature change of 3.2~4.8 °C, whereas those with ethylene-vinyl acetate (EVA) foam exhibited the lowest temperature change of −0.1~2.0 °C. Similarly, the samples with only TPU showed the greatest humidity change of −0.7~−5.5%RH. Moreover, the hole size had a larger effect on humidity change than material type. The samples with large holes achieved the largest humidity change of −4.4%RH, whereas the samples without holes had the smallest humidity change of −1.5%RH after 10 min (*p* < 0.001). Based on these results, various combinations of materials and hole sizes should be considered to fit the purpose of 3D printing clothing.

## 1. Introduction

Various ergonomic aspects should be considered when developing industrial protective clothing, sports protective clothing, and sports clothing [1]. In particular, protective clothing should be able to protect the human body from physical and toxic dangers, such as impact, abrasion, and flames [2,3]. Although sports clothing should also be used to protect the body from physical danger, its primary objective is to enhance athletic performance. To achieve this, enhancing comfort and reducing heat stress by effectively releasing sweat from the skin should be considered in fabricating clothing [4]. As such, basic research on the efficient use of ergonomics for new technologies and materials applied to clothing is essential.

Fused deposition modeling (FDM) 3D printing technologies have revolutionized product manufacturing and garnered attention across various fields over the last 40 years owing to their advantages of cost efficiency and ease of production [5]. After an attempt to commercialize 3D printing through open-source initiatives in 2005, the cost of 3D printers and filaments decreased rapidly, leading to its widespread use. For example, in 2013, Nike introduced shoes with 3D-printed outsoles for football players, reducing stress on the legs and enhancing performance by significantly reducing the weight of the shoes [6]. Additionally, in the medical field, 3D printing is used to produce implants and treat fractured bones and weakened joints. Furthermore, studies on organ creation using 3D bioprinting of live cells are being actively conducted [7].

In the clothing industry, which requires specific materials because clothing touches the skin directly, various 3D-printed filaments are being actively developed. In particular, 3D printing has been applied for protective clothing. Owing to its ability to produce customized garments using 3D scanning and design technologies, 3D printing is considered highly flexible compared to traditional manufacturing methods, especially for producing protective equipment [8]. Despite these advancements, FDM 3D printing for clothing still has limitations because of its reliance on thermoplastic filaments. In addition, it has a higher cost and longer processing time than general clothing manufacturing because it requires special equipment and professional skills [9]. Furthermore, 3D-printed clothing can provide limited comfort to the wearers due to its materials. The low flexibility of 3D-printed clothing owing to the morphological characteristics of 3D printing can also restrict movements.

Weaving methods can create micro holes in clothing materials to improve their flexibility and breathability, which helps in releasing heat and moisture from the skin. However, as 3D-printed clothing does not have pores that are significantly associated with breathability, it hinders the release of heat and moisture, thereby reducing the comfortable environment between clothing and skin. Therefore, in addition to the required functionality, the wearing and thermal comfort of 3D-printed cloths should be enhanced. Koo and Lee [10] invented wrist braces that are easily detachable with a grid structure, resulting in good breathability; however, the hardness and stiffness of polylactic acid (PLA) remains a challenge in ensuring the comfort of the wearer. To address these problems, thermoplastic polyurethane (TPU) filaments, which are known for their high flexibility, are increasingly used in 3D-printed protective clothing. Moreover, new applicable materials, such as thermoplastic elastomers (TPEs) with greater stretchability, have been developed [11]. Hong and Lee [12] developed hip protectors for snowboarding using TPU, and Jung et al. [13] invented 3D knee protectors for yoga by combining TPU and ethylene vinyl acetate (EVA) foam. These studies primarily focused on the development of protective clothing using TPU filaments and evaluating the wearing comfort of protective clothing. Furthermore, studies have been conducted on morphological development, including the fabrication of individualized chest protectors for hockey players by modeling human body data [14], fall-impact protection pads [15], and prototype crotch protectors for cycling [16].

The physical characteristics of the materials used in protective clothing and sportswear are currently analyzed. Mian et al. [17] analyzed and compared the mechanical, physical, and dimensional properties of acrylonitrile butadiene styrene (ABS), polyethylene terephthalate glycol (PETG), TPU, and polypropylene (PP), which are commonly used as FDM 3D printing filaments, to suggest appropriate materials for knee braces. Ronca et al. [18] compared the thermal, mechanical, rheological, and morphological characteristics of PLA and PETG to select an appropriate material for a scoliosis back brace. Additionally, to develop 3D-printed products in the form of assemblies or fabrics, Kim and Kim [19] and Lee [20] evaluated tensile strength, flexural strength, and laundry performance and conducted studies to verify the functional protective performance of fall-impact protection pads through impact testing [15] and to evaluate the differences in compression energy absorption and recovery capabilities [21]. However, enhancing thermal comfort inside clothing, among the various performance evaluations of 3D printing, has not been investigated in depth. As mentioned previously, clothing comfort is determined by the effectiveness of heat and moisture transmittance. Although this is an important factor from an ergonomic perspective, maximizing the transmittance remains difficult because of its multifactorial and dynamic mechanisms.

Therefore, this study measured temperature and humidity changes using EVA foam materials, which can enhance user mobility, and flexible TPU materials for use in 3D printing clothing development. Additionally, methods to efficiently release thermal energy and moisture were analyzed by measuring temperature and humidity changes based on the hole size, thereby establishing foundational data for the effective integration of 3D printing into clothing.

## 2. Materials and Methods

**Figure 1:**
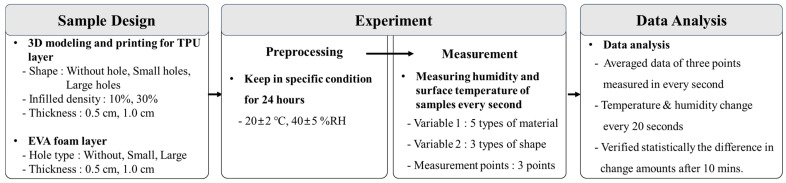
Procedures of the experiment.

Temperature and humidity changes were measured for 10 min for different hole sizes and materials to generate basic data for applying 3D printing to clothing. The process flow of the study is illustrated in Figure 1. Samples were modeled using 3D design software (TinkerCAD from Autodesk, Inc., San Rafael, CA, USA), printed using a 3D printer, and combined with EVA foam to produce 15 samples. All the samples were kept at a constant temperature of 20 ± 2 °C and 40 ± 5%RH (relative humidity) for 24 h prior to the experiment to control external factors. Each sample was placed on a hot plate, and its surface temperature and humidity were measured at three points every 1 s for 10 min. The results were analyzed by examining the changes in temperature and humidity every 20 s after 1 min. Furthermore, a statistical analysis was conducted to determine whether there were significant differences in the temperature and humidity changes depending on the variables after 10 min.

### 2.1. Experimental Variables and Samples

The experimental variables were three types of 3D printing hole sizes and five types of materials (Table 1). The hole size variable (variable 1) was selected to identify the most efficient structure for heat and moisture transfer of 3D-printed clothing. The hole sizes were categorized into three types: no holes, small holes, and large holes. The small holes comprised 49 holes with a radius of 2.5 mm, occupying 11.09% of the total area, whereas the large holes consisted of 36 holes with a radius of 4.0 mm, occupying 22.33% of the total area.

For the second variable, five materials were obtained by combining TPU with the infill densities of 10% and 30%, which are commonly used as clothing materials in 3D printing, and EVA foam, which is primarily used as protective padding material in clothing. The thickness of each sample was set to 1 cm, whereas that of the heterogeneous samples consisting of EVA foam and TPU were both set to 0.5 cm.

The 3D model of the TPU layer was created using the TinkerCAD (Autodesk, Inc., San Rafael, CA, USA) 3D design program and printed using the Original Prusa i3 MK3S+ 3D printer (Prusa Research, New York, NY, USA). Specifically, the printer applied the FDM (fused deposition modeling) method and was operated under the following conditions: nozzle temperature of 230 °C, bed temperature of 65 °C, layer height of 0.2 mm, nozzle diameter of 0.4 mm, outer border printing speed of 30 mm/s, and infill printing speed of 60 mm/s. Furthermore, a TPU filament (Cubicon Co., Seongnam, Gyeonggi-do, Republic of Korea) with a printing temperature of 210~235 °C, heat deflection temperature of 85~110 °C, and filament diameter of 1.75 mm (±0.05 mm) was used.

The mechanical properties, thermal conductivity, and moisture permeability of the samples used in this study are important factors that affect temperature and humidity changes. The tensile strength (ASTM D638 [22]) and flexural strength (ASTM D790-99 [23]) of the samples were measured with the ASTM method, and the results are listed in Table 2. Materials without holes had greater tensile and flexural strengths than those with holes. TPU with 30% infill density had the highest tensile and flexural strengths, whereas EVA foam had the lowest strength. Moreover, the thermal conductivity of TPU was 0.21~0.22 W/(m·K), and that of EVA foam was 0.030~0.046 W/(m·K) [24,25,26,27]. Furthermore, the moisture permeability of TPU was 10,139~10,428 g/(m^2^·24 h), whereas that of EVA foam was 27.6 g/(m^2^·24 h) [28,29].

### 2.2. Experimental Equipment and Methods

The experiment was conducted at a temperature of 34.5 ± 2 °C and humidity of 25 ± 5%RH. Prior to the experiment, all samples were stored at 20 ± 2 °C and 40 ± 5%RH for 24 h. The samples were placed on a hot plate (HP180D, Misonix Scientific Equipment Co., Seoul, Republic of Korea) with a temperature of 36.0 °C, which is similar to human skin temperature, to mimic actual clothing applications. The surface temperature and humidity of the samples were measured using a portable skin temperature and humidity sensor (Thermistor, LT-8B, Gram Co., Kyoto, Japan), with a measurement temperature range set from 0 to 70 °C. The surface temperature and humidity of each sample were measured every second for 10 min at three points, as listed in Table 3. For samples with holes, the measurements were obtained above the holes at three points.

The average values of the temperature and humidity changes at every 20 s timepoint from the three measurement points were used for the analysis. Considering that it takes time for the heat from the hotplate to be transferred to the surface of the samples, the temperature and humidity changes depending on the variables were analyzed starting at 60 s after the start of the experiment. Furthermore, descriptive statistics, one-way analysis of variance (ANOVA), Duncan’s post hoc analysis, and Bonferroni’s post hoc analysis were performed using IBM SPSS, Version 22 Statistics to determine the statistical difference in the temperature and humidity variations with the variables after 10 min.

## 3. Results

### 3.1. Heat and Humidity Transfer Based on Hole Size

One-way ANOVA was performed to analyze the differences in the temperature and humidity of the samples with different hole sizes (variable 1) after 10 min. As shown in Table 4, no statistically significant difference in the temperature change is observed after 10 min. However, statistically significant differences are observed in the humidity changes after 10 min (*p* < 0.01), so Duncan’s post hoc analysis was conducted. The results show that the largest humidity decrease of 4.4%RH is noted for the samples with large holes, followed by 2.9%RH for the samples with small holes, and finally, 1.5%RH for the samples without holes (*p* < 0.01). This phenomenon is attributed to the evaporation of moisture from the materials over time due to heat generation, whereby larger holes result in more moisture transferred. Therefore, it is expected that designing shapes with larger holes is expected to improve the comfortable environment between the clothing and skin. However, as no differences were observed in the temperature changes with different hole sizes after 10 min, further investigation into the temperature changes based on the material properties is needed. Additionally, analyzing changes over time, not just after 10 min, may be needed to fully understand the changes in temperature and humidity. This result is similar to the research conducted by Mazari et al. [30], which examined the moisture permeability of polyurethane (PU) foam samples with thicknesses of 60 mm and 85 mm and various hole sizes (hole diameter (mm): 0, 10, 15, 20) using the Standard Test Methods for Water Vapor Transmission of Materials (ASTM E96 [31]). Their research found that the PU samples with the maximum air area (area of the holes) have the highest moisture permeability, which is the same as the result of this research.

### 3.2. Temperature Change Depending on the Materials

To examine whether there were statistical differences in the temperature changes depending on the material (variable 2: EVA foam, TPU(10), TPU(30), EVA&TPU(10) and EVA&TPU(30)) with different hole structures (variable 1: without holes, with small holes, and with large holes) they were analyzed at every 20 s timepoint from 60 s to 10 min.

**Figure 2:**
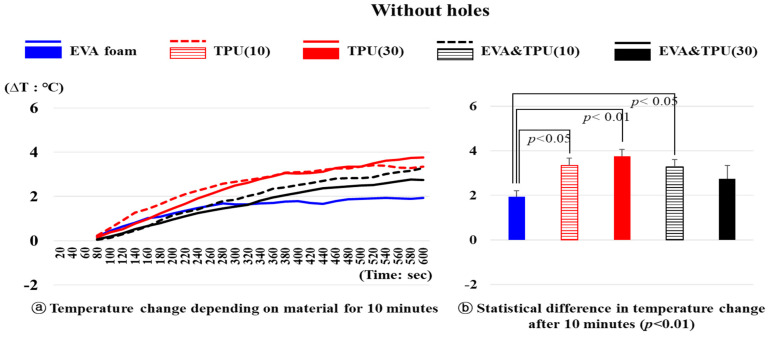
The temperature change of materials without holes and the statistical difference in temperature change after 10 min.

Temperature changes over time for the five materials (variable 2) without holes are shown in Figure 2. A statistically significant difference was observed in the temperature change depending on the material after 10 min (*p* < 0.01). Specifically, the temperature of EVA foam increased by 1.94 °C from 24.70 °C after 1 min, whereas that of TPU(10) increased by 3.35 °C from 23.93 °C (*p* < 0.05) and that of TPU(30) increased by 3.76 °C from 23.59 °C, showing a significant difference from EVA foam (*p* < 0.01). Additionally, EVA&TPU(10) exhibited a significant temperature increase of 3.28 °C from 23.57 °C compared to EVA foam (*p* < 0.05). More specifically, TPU(10) exhibited a rapid initial increase, reaching 2.57 °C at 280 s, followed by a steady increase until reaching its peak temperature change at 520 s. TPU(30) displayed a more gradual initial increase compared to TPU(10). However, at 340 s onwards, it exhibited a similar temperature change rate to TPU(10), with a steady increase until the highest temperature change of 3.76 °C after 600 s. The temperature changes for EVA&TPU(10) and EVA&TPU(30) were more gradual than for those materials made solely of TPU. Specifically, the temperature increases of EVA&TPU(10) and EVA&TPU(30) were 3.28 °C and 2.76 °C, respectively, after 10 min. Unlike other materials whose temperature increased steadily, no significant increase is noted for EVA foam after recording a temperature increase of 1.69 °C at 280 s. In particular, the maximum temperature increase of 1.94 °C after 600 s was obtained for EVA foam, which was the lowest temperature change among the materials tested.

**Figure 3:**
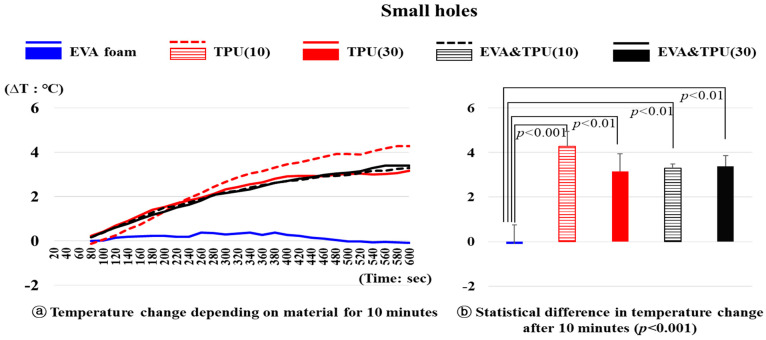
The temperature change of materials with small holes and the statistical difference in temperature change after 10 min.

The temperature changes over time for the five samples (variable 2) with small holes are depicted in Figure 3. After 10 min, there were statistically significant differences in the temperature changes depending on the materials (*p* < 0.001). EVA foam exhibited significantly lower temperature changes than the other materials. Specifically, EVA foam exhibited a slow temperature increase from 25.52 °C at 60 s to 0.38 °C at 380 s, followed by a slight decrease. In contrast, TPU(30), EVA&TPU(10), and EVA&TPU(30) showed similar trends, with the temperatures steadily increasing but exhibiting significantly higher temperature changes after 10 min compared to EVA foam (*p* < 0.01). After 10 min, the temperature of TPU(30) increased by 3.16 °C from 25.12 °C, that of EVA&TPU(10) increased by 3.30 °C from 23.42 °C, and that of EVA&TPU(30) increased by 3.39 °C from 28.84 °C. Additionally, TPU(10) showed the steepest temperature increase curve compared to the other materials, increasing by 4.28 °C from 23.45 °C after 10 min, which was significantly different from that of EVA foam (*p* < 0.001). In contrast, no statistically significant differences in temperature changes are noted for TPU(10), TPU(30), EVA&TPU(10), and EVA&TPU(30) materials.

**Figure 4:**
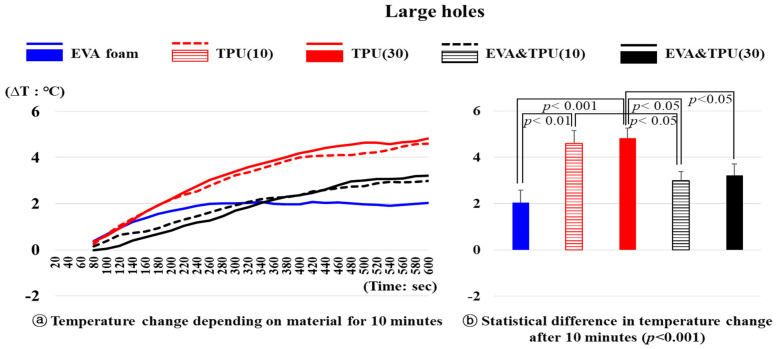
The temperature change of materials with large holes and the statistical difference in temperature change after 10 min.

The results of the temperature changes for the five samples (variable 2) with large holes are shown in Figure 4. The temperature changes depending on the material after 10 min were significantly different (*p* < 0.001). Specifically, the temperature increase of TPU(10) was 4.60 °C, whereas that for EVA foam was significantly lower at 2.04 °C (*p* < 0.01), and EVA&TPU(10) also exhibited a lower increase of 2.99 °C compared to TPU(10) (*p* < 0.05). Additionally, TPU(30) showed an increase of 4.82 °C after 10 min, which was significantly higher than that of EVA foam (*p* < 0.001), EVA&TPU(10), and EVA&TPU(30) (*p* < 0.05).

Among the samples with large holes, EVA foam exhibited the smallest temperature changes, with an increase of 2.04 °C from 24.41 °C after 10 min, followed by a rapid change until 260 s (1.99 °C increase), with slight fluctuations thereafter. The temperature of EVA&TPU(10) increased by 2.99 °C from 24.12 °C, whereas that of EVA&TPU(30) increased by 3.22 °C from 24.26 °C. Initially, EVA&TPU(30) displayed a more rapid temperature change than EVA&TPU(10); however, after 320 s, they exhibited a similar gradual increase. TPU(10) and TPU(30) exhibited similar changes, with both showing a relatively large temperature increase until approximately 400 s. TPU(30) exhibited slightly larger temperature changes than TPU(10). Ultimately, the temperature of TPU(10) increased by 4.60 °C from 23.55 °C after 10 min, while TPU(30) increased by 4.82 °C from 23.17 °C.

In summary, significant differences in temperature changes were observed depending on the material, which was an EVA foam, TPU, or a combination of both. Similarly, the trends in temperature changes varied accordingly. These material differences manifested differently depending on the shape of the material. In cases without holes, the temperature changes of EVA foam are significantly different from that of the other materials. More pronounced differences were observed in the samples with small holes. Moreover, for the cases with large holes, the differences in the temperature change trends based on the material were prominently displayed. This result is similar to the research of Eom et al. [32], which found that the shape and thickness of spacers of 3D printing affect thermal characteristics in cold environments. Particularly, as the hole size increased, the heat transfer to the surface got faster, and the temperature of the surface was measured to be high when the thickness of the spacer was 0.5 cm. Furthermore, the temperature change depending on EVA foam and TPU materials is similar to the result of a study by Lee et al. [33]. According to their result, EVA foam had the least temperature change, while TPU had the greatest. This tendency is related to thermal conductivity; the thermal conductivity (KS K 0466) of EVA foam was 0.030~0.046 W/(m·K) while that of TPU was 0.22 W/(m·K), which means that heat is transferred better in TPU than EVA foam [24]. Additionally, Girardin et al. [34] measured the thermal conductivity of EVA at temperatures ranging from ambient to 700 °C. The thermal conductivity of EVA measured from room temperature to 250 °C was found to be low, ranging from 0.0012 to 0.0032 W/(m·K). On the other hand, Pinedo et al. [35] measured the thermal conductivity of TPU at temperatures ranging from 0 to 130 °C. The thermal conductivity of TPU measured at room temperature (25 °C) was 0.15 W/(m·K), which is significantly higher than that of EVA foam. This indicates that TPU would be more effective in making comfortable clothing that requires rapid heat dissipation due to its higher thermal conductivity.

### 3.3. Humidity Change Depending on the Material

**Figure 5:**
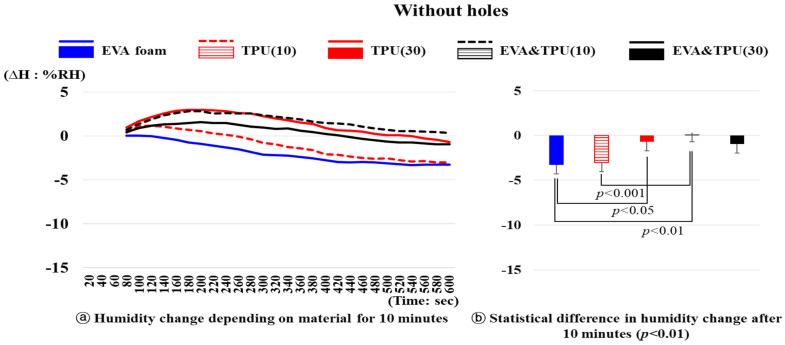
The humidity changes of materials without holes and the statistical difference in humidity change after 10 min.

The changes in humidity over time for five samples (variable 2) without holes are depicted in Figure 5. After 10 min, the humidity changes depending on the material were significantly different (*p* < 0.01). EVA foam exhibited a significantly larger decrease in humidity than TPU(30) (*p* < 0.05) and EVA&TPU(10) (*p* < 0.01). Moreover, TPU(10) showed a greater decrease in humidity than EVA&TPU(10) (*p* < 0.01).

The humidity of EVA foam gradually decreased from 31.50%RH at 1 min to 28.22%RH after 10 min, showing a decrease of 3.28%RH. In contrast, the humidity of the other materials increased initially and then decreased. For TPU(10), the humidity increased from 31.25%RH after 1 min to a maximum increase of 1.17%RH before decreasing to 28.24%RH after 10 min. Meanwhile, the humidity of TPU(30) showed an increase from 31.95%RH after 1 min to the maximum value of 2.96%RH before gradually decreasing to 31.25%RH after 10 min, indicating a decrease of 0.70%RH. The maximum increase in the humidity of EVA&TPU(10) exhibited a maximum increase of 2.81%RH before decreasing to 32.05%RH, while that of the EVA&TPU(30) was 1.54%RH before decreasing to 32.10%RH. The humidity of the materials, except for EVA foam, initially increased and then gradually decreased.

**Figure 6:**
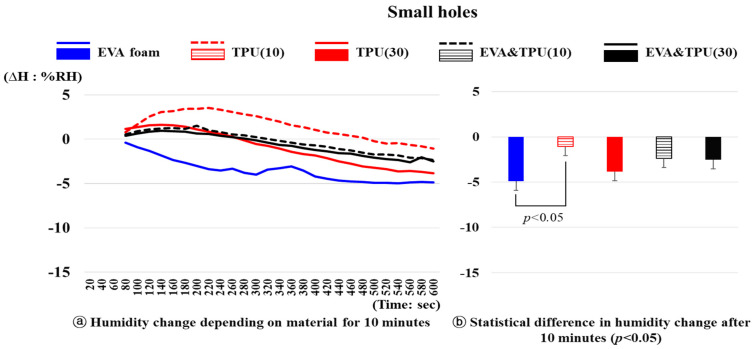
The humidity changes of materials with small holes and the statistical difference in humidity change after 10 min.

The changes in the humidity over time for the five samples with small holes (variable 2) are presented in Figure 6. A significant difference in the humidity change depending on the material was observed after 10 min (*p* < 0.05). A significant difference was noted between EVA foam and TPU(10) only (*p* < 0.05). Specifically, the humidity of EVA foam sharply decreased from 34.7%RH at 1 min to 29.79%RH after 10 min, resulting in a decrease of 4.91%RH, whereas that of TPU(10) initially increased by 3.51%RH from 30.80%RH at 1 min, and then decreased to 29.72%RH after 10 min, representing a decrease of 1.07%RH. For TPU(30), EVA&TPU(10), and EVA&TPU(30), the humidity increase was not substantial, ranging from 0.92%RH to 1.60%RH, gradually increasing before decreasing. In particular, after 10 min, the humidity of TPU(30) (max: 1.60%RH) decreased from 32.83%RH to 3.84%RH after 10 min, that of EVA&TPU(10) (max: 1.50%RH) decreased from 32.49%RH to 2.38%RH, and that of EVA&TPU(30) (max: 0.92%RH) decreased from 33.73%RH to 2.52%RH.

**Figure 7:**
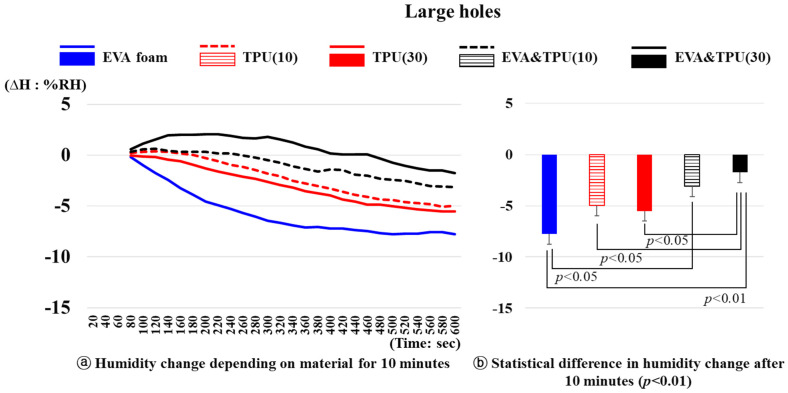
The humidity changes of materials with large holes and the statistical difference in humidity change after 10 min.

The changes in humidity over time for the five materials with large holes (variable 2) are shown in Figure 7. After 10 min, a significant difference in the humidity changes of the different materials is noted (*p* < 0.01). Specifically, the humidity change of EVA&TPU(30) was significantly lower than that of EVA foam (*p* < 0.01), TPU(10) (*p* < 0.05), and TPU(30) (*p* < 0.05). Notably, for EVA&TPU(30), the humidity initially increased by 2.05%RH from 31.92%RH at 1 min and then decreased to 1.76%RH after 10 min. In contrast, the humidity change of the other samples decreased without an initial increase. Furthermore, for EVA&TPU(10), the humidity change was significantly lower than that of EVA foam (*p* < 0.05). Specifically, after 10 min, the humidity of EVA foam (max: −0.38%RH) decreased by 7.75%RH from 36.21%RH after 10 min, that of TPU(10) (max: 0.40%RH) decreased by 4.97%RH from 32.90%RH, and that of TPU(30) (max: −0.04%RH) decreased by 5.51%RH from 33.02%RH. For EVA&TPU(10) (max: 0.66%RH), the humidity decreased by 3.12%RH from 32.69%RH at 1 min to 29.67%RH after 10 min.

In other words, although the humidity trends among the samples were similar regardless of hole size, the trend became more pronounced as the hole size was larger. Examining these trends, EVA foam showed the largest humidity change, followed by TPU(30), TPU(10), EVA&TPU(10), and EVA&TPU(30), which had the smallest humidity change. These results are similar to the result of a study by Lee et al. [33], which found that the humidity of EVA foam exhibited the largest decrease. In contrast, samples that contained TPU showed a trend of increasing surface humidity at the beginning and decreasing as time passed. The reason why only EVA foam has different trends can be explained by the material’s properties. Examining the thermal conductivity and moisture permeability, TPU had a moisture permeability of 10,139~10,428 g/(m^2^·24 h) [28] and a thermal conductivity of 0.22 W/(m·K), while EVA foam had a moisture permeability of 27.6 g/(m^2^·24 h) [29] and a thermal conductivity of 0.030~0.046 W/(m·K), which was lower than TPU. Therefore, the lower thermal conductivity and moisture permeability of EVA foam result in a slower transfer of heat from the heat source to the sample surface. This gradual temperature increase leads to a slower release of the moisture contained within the sample, causing the humidity variation to be the greatest after 10 min. Also, to apply the result of this research to the development of various sportswear or protective clothing, physical properties, such as tensile strength and flexural strength, should be considered as well. Examining the tensile and flexural strength measured in the research, the common EVA foam used in clothing exhibits the lowest values among the materials, with a tensile strength ranging from 0.6 to 1.1 MPa and a flexural strength ranging from 3.9 to 4.1 MPa. In contrast, TPU(30) showed significantly higher values, with a tensile strength ranging from 5.0 to 8.6 MPa and a flexural strength ranging from 28.1 to 30.5 MPa. And both tensile and flexural strength were lower when holes were present in the sample compared to samples without holes. Therefore, when applying to 3D-printed summer sportswear, which requires heat and sweat dissipation, applying TPU(10) or TPU(30), which have high moisture permeability and thermal conductivity, would be good. However, when clothing comfort is a priority over dissipating sweat, applying EVA&TPU(10) or EVA&TPU(30) would likely be more optimal since TPU has high flexural strength, which can result in discomfort and limitation of movement.

## 4. Discussion and Conclusions

This study measured and analyzed the changes in temperature and humidity based on the material, that is, TPU, EVA foam, and their combination, and hole size, that is, without holes and with small and large holes, for 10 min to suggest an effective method for applying 3D printing to clothing while maintaining a comfortable inner environment between the clothing and skin.

The samples with large holes exhibited the greatest humidity change, whereas those without holes exhibited the smallest change after 10 min. This indicates that 3D printing should be modeled with large holes when used in sports or summer clothing to ensure comfortable inner clothing. Furthermore, the changes in temperature depending on the material (variable 2: EVA foam, TPU(10), TPU(30), EVA&TPU(10), and EVA&TPU(30)) were analyzed. EVA foam with small holes or without holes displayed a minimal change in temperature, whereas the other four materials with TPU exhibited significant changes in temperature. Furthermore, the temperature of samples with only EVA foam with large holes initially had a rapid change in temperature, which then slowed down. On the other hand, samples of TPU only (i.e., TPU(10) and TPU(30)) with large holes had continuous rapid changes. Eom et al. [32] achieved similar results in the analysis of heat characteristics depending on the spacer shape used for 3D printing in cold environments. In particular, the spacer with a thickness of 0.5 cm exhibited faster heat transfer with larger holes. Therefore, it can be concluded that 3D-printed clothing made of TPU with large holes is optimal for quickly dissipating body heat. It is estimated that TPU conducts heat more effectively than EVA foam since TPU has a higher thermal conductivity of 0.22 W/(m·K), while EVA foam’s thermal conductivity is 0.030~0.046 W/(m·K) [24,25,26,27]. This indicates that thermal conductivity is closely related to the change in the temperature of the material. Therefore, materials with high thermal conductivity are suitable for summer clothes or sportswear, which are important for decreasing the stress caused by heat, while materials with low thermal conductivity are suitable for winter clothes, which are important for maintaining heat. Furthermore, having more and larger holes would be beneficial for situations where the release of sweat from the human body is important.

Specifically, in the aspect of the change in humidity, while EVA foam was the only sample that showed a significant difference in the category of small holes or without holes, there was a significant difference between materials in the category of large holes. EVA foam achieved the largest change in humidity, followed by the samples with only TPU (TPU(10) and TPU(30)). Meanwhile, the samples of combined TPU and EVA foam displayed minimal changes in humidity. However, the reason for the significant change in the humidity of EVA foam is that the EVA foam contains more moisture than the 3D-printed TPU samples, resulting in more ejection of moisture. In addition, as EVA foam has a low thermal conductivity, the slow heat transfer from the hot plate to the surface of EVA foam affected the moisture transfer, resulting in the largest change in humidity after 10 min. Meanwhile, the moisture in the TPU samples was transferred with heat from the hot plate to the surface, resulting in the humidity increase at the beginning but followed by gradual changes due to the small amount of inner moisture remaining after evaporation. Consequently, the lowest humidity change was obtained after 10 min. 

The amount of change in humidity is likely related to moisture permeability. The moisture permeability of TPU is 10,139~10,428 g/(m^2^·24 h), which is significantly higher than that of EVA foam (27.6 g/(m^2^·24 h)) [28,29]. Therefore, it is expected that applying TPU samples to clothing would be more efficient for sweat evaporation from the skin compared to EVA foam. In addition to the moisture permeability, the moisture absorption of the material is considered an important physical property. Specifically, the size of the holes is considered an important factor that has a significant impact on the change in humidity than temperature. Therefore, structures with holes should be used in sportswear and high-strength protective clothing, which are often exposed to sweat.

In general, industrial and sports protective clothing must be developed by considering various ergonomics aspects [1]. Recently, 3D-printed clothing has been increasingly used in protective clothing and sportswear owing to its advantages in terms of thickness and shape retention. Thus, physical performance, such as impact resistance and abrasion, should be ensured while maintaining comfort and reducing heat stress to enhance athletic performance [4]. Therefore, the comfort of 3D-printed clothing can be improved if the change in temperature and humidity, as obtained in this study, and the physical properties of the material, such as thermal conductivity and moisture permeability, are comprehensively considered. 

However, the strength, including the tensile strength and flexural strength, as well as impact resistance and other functional aspects of sportswear, must be ensured before applying 3D printing to protective clothing. According to the tensile strengths and flexural strengths measured in this study, the tensile strength and flexural strength of standard EVA foam, which is commonly used in clothing, were found to be the lowest, ranging from 0.6 to 1.1 MPa and 3.9 to 4.1 MPa, respectively. On the other hand, TPU(30) exhibited significantly higher values, with tensile strength ranging from 5.0 to 8.6 MPa and flexural strength from 28.1 to 30.5 MPa. When TPU and EVA foam were combined, the values were intermediate, with tensile strength ranging from 2.7 to 4.8 MPa and flexural strength from 13.0 to 13.6 MPa. Therefore, due to its high tensile strength, TPU is suitable for clothing that requires protective functions. However, because the flexural strength of TPU is also high, it is recommended to use TPU only in certain parts of clothing where flexibility and mobility are necessary. These measurement results are consistent with the findings of previous studies [36,37,38,39].

Evaluating the tensile strength, flexural strength, and impact resistance of EVA foam, TPU, and EVA&TPU samples, EVA foam has the lowest flexural strength and low tensile and impact resistance, meaning that it is highly flexible but adequately unprotectable from external impact. In contrast, TPU achieved high tensile strength, impact resistance, and flexural strength, denoting its strength against external impact but low flexibility. Samples of combined TPU and EVA foam achieved higher tensile strength and impact resistance than EVA foam and lower values than TPU but with good flexural strength at the same time. Thus, the combined sample of TPU and EVA foam can realize improved comfort for wearers, highlighting its potential as a good material for clothing worn directly on the skin even though it has a lower tensile strength and impact resistance than TPU. As larger holes decrease impact resistance [33], a suitable hole size should be fabricated depending on the importance of protection from external impacts and the comfort of the clothing. Considering the importance of impact resistance, 3D-printed protective clothing of TPU is the most appropriate, followed by the combinations of TPU and EVA foam. Particularly, when making a protector for athletics, a material with large holes instead of without holes can maintain a comfortable environment inside the clothing. Furthermore, increasing the thickness is another approach to enhance impact resistance.

However, further research is required to determine the extent of comfort of 3D-printed TPU protective clothing. In addition, when 3D-printed products are applied to clothing, the changes in temperature and humidity would differ depending on the properties of the fabric cover. Therefore, further research on temperature and humidity changes depending on covers with different properties should be conducted to collect data for practical clothing applications. The conclusions of this study will not only improve the comfort and function of temperature and humidity of 3D-printed clothing but will also be used as fundamental data for efficiently incorporating 3D printing into clothing.

## Figures and Tables

**Table 1:** Experimental variables.

	Materials	EVA Foam	TPU(10)/TPU(30)	EVA&TPU(10)/ EVA&TPU(30)	Modeling
Hole Sizes					
No holes		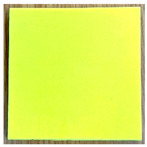	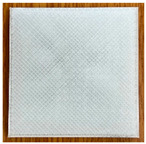	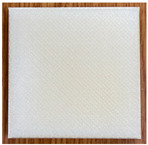	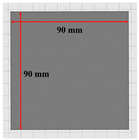
Small holes		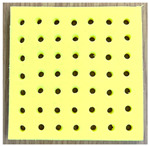	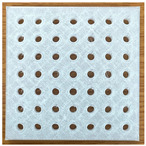	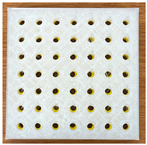	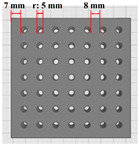
Large holes		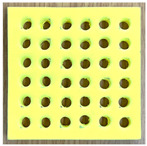	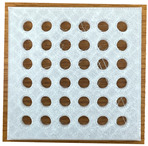	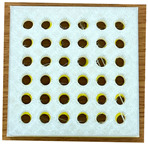	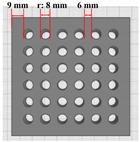
Side view		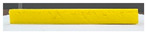	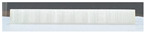	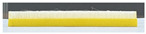	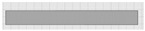

**Table 2:** Tensile and flexural strengths of different materials.

	Materials	EVA Foam		TPU(10)		TPU(30)		EVA&TPU(10)		EVA&TPU(30)	
Properties		without Holes	with Holes	without Holes	with Holes	without Holes	with Holes	without Holes	with Holes	without Holes	with Holes
Tensile strength (MPa): ASTM D638(2014) ^(a)^		1.1	0.6	6.8	3.7	8.6	5.0	4.5	2.7	4.8	2.8
Flexural strength (MPa): ASTM D790-99 ^(b)^		4.1	3.9	25.4	24.1	30.5	28.1	13.6	13.5	13.6	13.0

(a) Test speed: 500 mm/min; (b) Test speed: 1 mm/min, span distance: 82 mm.

**Table 3:** Measurement equipment and locations.

Measurement Equipment		Measurement Locations	
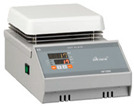	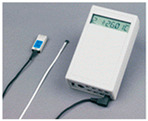	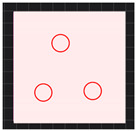	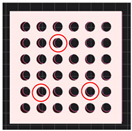
<Hot plate>	<Surface temp. & humidity>	<Without holes>	<With holes>

**Table 4:** Temperature and humidity changes after 10 min depending on hole sizes.

		without Holes	Small Holes	Large Holes	F	*p*
∆T (Temperature change after 10 min, °C)	Mean	3.0	2.8	3.5	1.389	0.261
	SD	0.7	1.6	1.2		
∆H (Humidity change after 10 min, %RH)	Mean	−1.5 ^c^	−2.9 ^b^	−4.4 ^a^	8.522	0.001
	SD	1.6	1.8	2.2		

Duncan post hoc analysis results: a < b < c.

## Data Availability

The original contributions presented in the study are included in the article, further inquiries can be directed to the corresponding authors.
